# Hawks, doves and ornithological aspirations—What bird should an OSCE examiner be?

**DOI:** 10.1111/medu.70049

**Published:** 2025-12-19

**Authors:** Stephen Meldrum

**Affiliations:** ^1^ Edinburgh Medical School University of Edinburgh Edinburgh UK; ^2^ School of Medicine University of Dundee Dundee UK

## Abstract

Calling all OSCE examiners… Are you a hawk or a dove? And, is there a better alternative? #MedEducUnleashed

*So, are you a hawk or a dove?*

It is a fascinating question—and a rather contentious one, too. The idea of describing people as hawks or doves seems to stem back to at least the War of 1812, when Americans in favour of military action against the United Kingdom were labelled ‘war hawks’, whereas anti‐war opposition were termed ‘doves’.

Nowadays, and certainly within clinical education, the terms are used to describe assessors who differ from one another in the stringency or leniency of their marking.^1^ It has become particularly common parlance within objective structured clinical examinations (OSCEs), where a high value is placed on inter‐rater reliability and standardisation.

Hawk examiners are stringent and difficult to impress. They are caricatured as being like birds of prey, looking for signs of weakness so that they can swoop. They wait, watch, hover and when a candidate makes a mistake or says the wrong thing, they will strike, scoring them down on their mark sheet.

Dove examiners are quite the opposite. They are lenient, non‐predatory, nice, soft and gentle. They are easier to please, and a candidate will not necessarily have to perform at a high standard to acquire a good mark from them.

Hawks and doves can both be problematic. If all examiners are hawks, then an assessment becomes very difficult to pass. If all examiners are doves, then an assessment becomes easy to pass—perhaps *too* easy.

Over the years, I have met OSCE examiners who seemed to proudly identify as either a hawk or a dove. Not just that, but it is even possible to harbour derogatory views about examiners on the other end of the ornithological spectrum. ‘I'm a hawk – those gentle doves might let anyone pass, but I'm doing my bit to maintain standards.’ ‘I'm a dove – those nasty hawks need to be nicer and remember that medical students aren't consultants.’

Thankfully, very few examiners are absolute hawks (looking for flawless performance before they award a pass mark) or absolute doves (awarding a pass mark for almost any level of performance, no matter how poor). In reality, an examiner may be slightly more ‘hawkish’ or slightly more ‘dovey’ than another examiner, but they can probably counteract each other over a whole OSCE assessment if there are enough stations and data collection points.

However, the concept of hawks and doves does raise an interesting thought—if examiners can be too much like hawks or too much like doves, what bird do we actually want them to resemble? In other words, as OSCE examiners, is there a bird that we could aspire to be like?

I have posed this question to various examiners in the past. The crow is a standard answer. However, although the crow certainly sits somewhere between hawk and dove on the predatory spectrum, there are problems with it. Generally speaking, crows do not have a great image—noisy, dirty, associated with scavenging, graveyards and death. As an examiner, it is not a good look. For the same reason, I do not think seagulls are an ideal choice either.

People also seem to like robins. However, aside from them being incredibly territorial, we tend to associate robins with a specific time of year. That is a potential problem for an OSCE examiner, who we would usually expect to be active throughout the year (whether that be in clinical practice, or teaching, or other areas of curriculum delivery), rather than only being visible during OSCE season.

So, if the crow, the seagull and the robin do not fit, is there perhaps another bird that does?

After giving it a bit of thought, I have identified three characteristics that I think our examiner‐bird should have ….

First, I think we should try to find a bird that is reasonably discrete. The focus of an OSCE station is the candidate, not the examiner. Examiners should not be the focus of attention because the exam is not about them. Swans are too big. Woodpeckers are too noisy. Macaws are too colourful. Peacocks are too flamboyant. We must find a bird that is happy to sit quietly, observe and will not be a distraction.

Second, I think we should try to find a bird that can thrive in a range of environments on a variety of foods. Likewise, OSCE examiners would usually be expected to have a degree of flexibility and adaptability, being able to assess different skills and different competencies within different simulated settings. An examiner who insists on assessing only one specific skill and absolutely nothing else is of limited utility.

Third, I think we should try to find a bird species whose males and females have similar markings. Within the bird kingdom, males and females of the same species often look very different. There are many wonderful examples of this, like the mallard duck, with its green‐headed males and brown‐headed females. There is also the pheasant, the blackbird, the whitethroat, the capercaillie and so on. However, within an OSCE, we should not be able to look at a candidate's score and know the demographics of the examiner, whether that be sex, age, ethnicity or anything else—marking needs to be the same.

Thus, after some exploration, I would like to offer you the goldfinch. It is a lovely bird, and I think we will be doing well as examiners if we can resemble it a little more.

Called *Carduelis carduelis* in Latin, it is native to Europe, Africa and Asia, but has also been introduced to North America, South America, Australia and New Zealand. Found across farmland, gardens and scattered trees, its preferred diet is thistle and weed seeds, but it will consume a variety of plant and animal matter when necessary, including buds, bark, sap, berries and insects.

An adult male stands about 12 cm tall, with a red face, brown and white body and black and yellow wings. An adult female also stands about 12 cm tall, with a red face, brown and white body and black and yellow wings. Marking is very similar.

In addition to these attributes, the goldfinch's song is beautiful. In fact, it inspired Vivaldi to compose *Il Gardellino*, a concerto during which the flute imitates the bird's sweet trills. When the goldfinch opens its mouth, it is worth listening to.

So, let us do away with hawks and doves and become more like goldfinches—not too strict, not too lenient, not the focus of attention, happy to assess a range of skills and similar to our fellow examiners in our marking.

Furthermore, a flock of goldfinches is called a ‘charm’.^2^ I reckon that should be our new collective noun for a group of OSCE examiners.

## CONFLICT OF INTEREST STATEMENT

The author declares no conflict of interest.

## Data Availability

The data that support the findings of this study are available on request from the corresponding author. The data are not publicly available due to privacy or ethical restrictions.
